# High preoperative blood levels of HE4 predicts poor prognosis in patients with ovarian cancer

**DOI:** 10.1186/1757-2215-5-20

**Published:** 2012-08-21

**Authors:** Grigorios Kalapotharakos, Christine Asciutto, Emir Henic, Bertil Casslén, Christer Borgfeldt

**Affiliations:** 1 Department of Obstetrics & Gynecology, Skanes University Hospital, Lund, Sweden; 2 Department of Obstetrics and Gynecology Region Skåne University Hospital, Lund University, SE-22185, Lund, Sweden

**Keywords:** HE4, ROMA, Ovarian neoplasm, Survival analyses, Prognosis

## Abstract

**Abstract:**

The aim of this study was to assess the clinical value of preoperative blood levels of HE4 as a predictor of overall survival in patients with ovarian cancer and to validate previous data of HE4 and the ROMA algorithm including HE4 and CA125 in discriminating benign and malignant ovarian tumors.

**Experimental design:**

The preoperative plasma levels of HE4 and CA125 were analyzed with ELISA in 312 patients with adnexal lesions. Tumors were classified as benign (n= 206), borderline (i.e. low malignant potential tumors) (n= 25), and well (n= 14), moderately (n= 15), and poorly (n= 51) differentiated malignant.

**Results:**

In univariate Cox regression analyses high levels (dichotomized at the median) of HE4, CA125, increased age (continuous variable), advanced-stage of disease 2–4, histological grade 3 and non-optimal tumor debulking at primary surgery were all significantly associated with shorter overall survival. A multivariate Cox regression model including pre-operative available covariates HE4 and CA125 both dichotomized at median in addition to age as continuous variable showed that high levels of HE4 was an independent prognostic marker for worse prognosis HR 2.02 (95% CI 1.1-3.8). In postmenopausal women the ROMA algorithm gave the highest AUC of 0.94 (95% CI, 0.90-0.97) which was higher than the separate markers HE4 AUC 0.91 (95% CI 0.86-0.95) and CA125 AUC 0.91(95% CI 0.87-0.96).

**Conclusions:**

High concentration of plasma HE4 is an independent preoperative marker of poor prognosis in patients with ovarian cancer. The algorithm ROMA discriminates in postmenopausal women between malignant and benign tumors with an AUC of 0.94.

## Introduction

The majority of patients with ovarian cancer are not diagnosed until the disease is in advanced stages due to mild and diffuse symptoms. These patients face poor prognosis since the five year overall survival is only 40–45%. In contrast, early-stage ovarian cancer i.e.(before the tumor has spread in the peritoneal cavity) has excellent curability. Numerous efforts have been done to identify a biomarker which will allow screening of population cohort at risk, but so far without substantial success. Even the most used tumor marker CA125, is not reliable due to low sensitivity in patients with early-stage ovarian cancer [1]. Also CA125 has low specificity since it is often increased in patients with benign endometriosis. Gynecologic ultrasound has high sensitivity and acceptable specificity but is too labor intense to be employed for screening. Thus a new tumor biomarker or a combination of biomarkers with high sensitivity and reasonable specificity for early stage ovarian cancer is urgently needed.

Such biomarker can hopefully be employed for screening of asymptomatic women in age group at risk in order to promote early detection and thus increase curability.

Human Epididymis Protein 4 (HE4) is a novel tumor marker approved by the United States FDA for monitoring recurrence or progressive disease in patients with epithelial ovarian cancer. HE4 is a secreted low molecular weight glycoprotein that is predominantly expressed in epithelial cells of the epididymis and the normal female reproductive tract. Although its physiological functions have not been fully identified, over expression of the HE4 protein has been found to occur in serous and endometrioid ovarian carcinomas [2].

Blood levels of HE4 reportedly have potential as biomarker for epithelial ovarian cancer. Moore et al. have published papers using the combination of CA125, HE4 and menopausal status to predict the presence of a malignant ovarian tumour in the Risk of Ovarian Malignancy Algorithm (ROMA).

The combination of HE4 and CA125 in the ROMA seems to have higher sensitivity than either biomarker alone [3,4]. Also combining HE4 and CA125 seems to discriminate more accurately ovarian cancer from ovarian endometriotic cysts.

Although HE4 offers a new tool in the management of women with pelvic masses, the association of preoperative blood levels of HE4 with outcome in patients with ovarian cancer remains to be defined. The aim of this study was first is to validate the previous data on discrimination of benign and malignant ovarian tumors and second to assess the clinical value of preoperative serum HE4 levels as a predictor of overall survival in patients with epithelial ovarian cancer.

## Materials & methods

### Patients

Peripheral blood samples were obtained preoperatively in 312 patients admitted for primary surgery of adnexal masses to the Department of Obstetrics and Gynecology in Lund 1993–2009. Blood was collected in citrate tubes, centrifuged, and the plasma stored at −20°C until analyzed. The laparoscopic or open surgical procedures in benign cases included resection of the cyst or unilateral oophorectomy, and in the malignant cases abdominal hysterectomy, bilateral salpingo-oophorectomy, and infracolic omentectomy and lymphadenectomy in the pelvis and para-aortic area when indicated. Cytologic analysis of ascitic fluid or when absent of peritoneal washing was performed. All diagnoses were verified by histopathology of the tumors. Histopathological grade and stage of the disease (FIGO) were available in all malignant cases. Postoperative adjuvant treatment was given according to clinical standards in patients with invasive cancer. Patients with stage Ic or higher stage received platinum based chemotherapy, either alone or combined with paclitaxel. Survival status of all patients, i.e. alive or dead including date of death was obtained on September 17, 2010 from the Swedish Population Register (Tumor registry center in Lund). Histopathological diagnoses, grade and stage of the disease (FIGO) were available in all malignant cases, as shown in Table 1 and 2. In patients with invasive tumors the median follow-up time after surgery was 31 months (range 0.2-203 months). The median follow-up time for patients alive was 90 months (range 19–203 months). The median age was 54.5 years in the entire material (range 16–90, mean 54.9 ± 16; SD), and 63.1 years in the sub-group of patients with invasive cancer (range31-87, mean 64.1 ± 12; SD).

**Table 1:** Histo-pathological diagnoses in relation to differentiation and grade of the ovarian tumor

**Differentiation (Grade)**	**Serous**	**Mucinous**	**Endometroid**	**Clear Cell**	**Teratoma**	**Endometriosis**	**Funtional**	**Total**
Benign	90	38	0	0	23	39	16	206
Borderline	14	10	1	0	0	0	0	25
Well G1	8	0	6	0	0	0	0	14
Moderately G2	11	1	3	0	0	0	0	15
Poorly G3	32	5	9	5	0	0	0	51
Total	155	54	19	5	23	39	16	311

**Table 2:** Histo-pathological grade differentiation in relation to stage

	**Stage**				
**Differentiation**	**1**	**2**	**3**	**4**	**Total**
Borderline	27	1	2	0	30
Well	14	2	5	2	23
Moderately	5	2	10	1	18
Poor	6	7	50	5	68
Total	52	12	67	8	139

The study was approved by the Review Board at the Faculty of Medicine, University of Lund, Sweden.

***CA125:*** Preoperative plasma samples were routinely assayed for CA125 using a commercial electro-chemo-luminescence immunoassay Elecsys CA125 kit™ (Roche). The assay was performed according to the manufacturer’s instructions.

***HE4***: The HE4 EIA assay (Fujirebio Diagnostics, Goteborg, Sweden) met the laboratory quality criteria, and quality criteria samples performed within acceptance limits. The inter-assay variation was 1.2% and the intra-assay of samples measured in duplicate was 3.0%. Clinical and histopathological data were not available to the technicians performing the assays. Plasma and serum were collected simultaneously and the levels of HE4 matched with an acceptable correlation.

ROMA (Risk of Ovarian Malignancy Algorithm) is a predicative probability algorithm that classifies women with pelvic mass or ovarian cyst as being at high or low risk for epithelial ovarian cancer [5].

This predicative probability algorithm is based on menopausal status and preoperative levels of HE4 and CA125 and is calculated as follows:

Predictive Index (PI) for premenopausal patients:

PI = −12.0 + 2.38 x ln(HE4) + 0.0626 x ln(CA125)

Predictive Index (PI) for postmenopausal patients:

PI = −8.09 + 1.04 x ln(HE4) + 0.732 x ln(CA125)

Predicted probability = exp(PI) / [1+ exp(PI)].

### Statistical methods

Differences between groups regarding plasma content of HE4 and CA125 were evaluated with the Mann–Whitney U test for unpaired samples, ANOVA with Bonferroni as post hoc test and trends across ordered groups were analyzed using linear regression with log transformed values. Spearmans rho was used as a measure of correlation between the parameters. Overall survival probabilities were calculated using the Kaplan-Meier method. The Cox proportional hazard model was used in univariate and multivariate survival analyses. Point estimates were reported as hazard ratios (HR) and 95% confidence intervals (CI). Assumptions of proportional hazards were verified graphically where applicable. Significant departures from proportionality were observed neither for dichotomized HE4 nor for other covariates used in the Cox regression analyses. All comparisons were two-sided, and a 5% level of significance was used. The statistical analyses were performed using SPSS™ (18.0.0).

## Results

**Figure 1:**
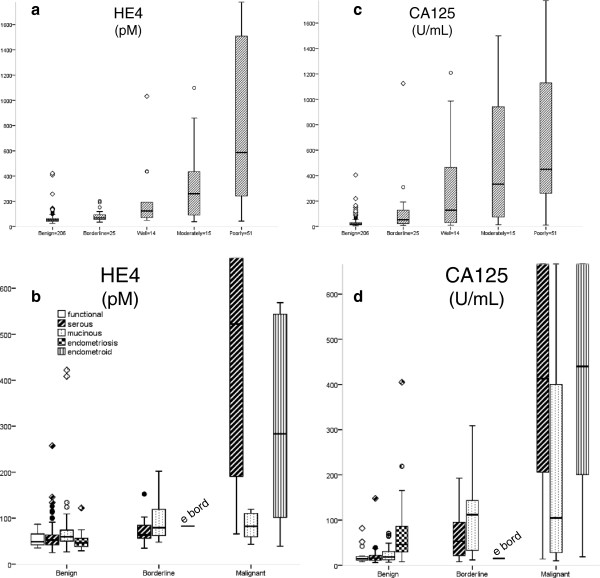
**a-d Peripheral blood concentrations HE4 and CA 125 obtained preoperatively in patients with adnexal lesions. **The boxes represent the 25^th^, 50^th^, 75^th^ percentiles. Bars include highest and lowest values, except outliers (○), which are 1.5 to 3 box lengths from the end of the box, and extremes (◊) which are more than 3 box lengths from the end of the box.

The plasma levels of pre-operative HE4 were higher in patients with borderline (p < 0.001) and invasive ovarian (p < 0.001) tumors than in those with benign tumors (Figure 1a). The median plasma level of HE4 for all patients with benign lesions was 52 pM (range 25–422) while the borderline group showed a median value of 69 pM (range 35–202, p <0.05 ; Table 3 and 4). Patients with mucinous benign lesions had slightly significant higher levels of HE4 (p < 0.05) compared with functional, serous and endometriotic ovarian cysts (Figure 1b). Patients with endometriosis had decreased levels of HE4 compared to patients with other benign cystic lesions (p = 0.04). The median of plasma HE4 concentrations in patients with endometriosis was 46 pM (range 29–122).

**Table 3:** The median plasma levels with correlating range for HE4 (pM) and CA 125 (U/ml) in benign patients

**Benign Group**	***HE4***		***CA 125***	
	***Median/Range***		***Median/Range***	
Endometriose (n = 39)	46	29 -122	46	8-4992
Functionell (n = 16)	48	35-87	14,5	8-82
Mucinous (n = 38)	59,8	26-422	18,5	7-71
Serous (n = 90)	52,1	25-258	16	6-148
Dermoid (n = 23)	47,6	30-141	19	6-151
Total benign (n = 206)	52	25-422	19	6-4992

**Table 4:** The median plasma levels with correlating range for HE4 (pM) and CA 125 (U/ml) in borderline patients

**Borderline Group**	***HE4***		***CA 125***	
	***Median/Range***		***Median/Range***	
Endometrioid (n = 1)	83	/	15	/
Mucinous (n = 11)	79	48- 202	112	12-309
Serous (n = 15)	68	35-152	53	8-1225
Total borderl (n = 27)	69	35-202	53	8-1225

Endometriosis was surgically staged according to the classification of the American Society of Reproductive Medicine (ASRM) [6,7]. In women with endometriosis HE4 levels increased in advanced clinical stage: ASRM stage I (n = 21): median 40 pM (range 29–69), ASRM stage II (n = 15): median 56 pM (range 29–75), ARSM stage III and IV (n = 3): median 73 pM (range 43–122, p < 0.003). The median HE4 levels were in all ASRM stages within the normal range. One patient with endometriosis had HE4 lever above 75 pM (Figure 1b).

The plasma levels of CA 125 were higher in patients with borderline (p < 0.001) and invasive (p < 0.001) tumors than in patients with benign tumors (Figure 1c). The CA 125 levels in the endometriosis group were higher than the levels in the remaining benign ovarian tumor group (p < 0,001), similar to those in the borderline group (p = 0.69), but lower than the malignant tumor group (p < 0.001 ; Figure 1c and d).

The ROC curves comparing benign to invasive tumors yields AUC values which are shown in Table 5. Since the menopausal status was unknown in several patients, the premenopausal age was set at 50 and postmenopausal age was set at 54 years or older. The median menopausal age is 51.8 in Sweden. Analyses with different menopausal cut off ages did not significantly change the AUC curves, using 48 and 56 or 51,8 as pre- and postmenopausal age differed in the AUC curves 0.01 from 50 and 54 year as cut off ages for both HE4 and CA125. The number of included women in the AUC analyzes excluding age 50–54 was 277 out of 312 (89%). In postmenopausal women the proposed ROMA score gave the highest AUC of 0.94 (95% CI, 0.90-0.97) which was higher than the separate markers HE4 AUC 0.91 (95% CI 0.86-0.95) and CA125 AUC 0.91(95% CI 0.87-0.96 ; Table 5). Also for stage I-II ovarian cancer including borderline tumors in postmenopausal women, the ROMA score showed the higher AUC 0.84 (95% CI, 0.75-0.92) than the separate markers HE4 AUC 0.82 (95% CI 0.74-0.91) and CA125 AUC 0.82 (95% CI 0.73-0.92). In premenopausal women the proposed ROMA score had AUC 0.73 (95% CI 0.58-0.88) which was similar to HE4 AUC 0.73(95% CI 0.58-0.87) but lower than CA125 AUC 0.82 (95% CI 0.73-0.92). When borderline tumors were excluded the ROMA AUC was 0.95 (95% CI 0.91-0.99) in postmenopausal women and the ROMA AUC was 0.83 (95% CI 0.65-1.00) in premenopausal women. The ROMA cut-off set at fixed specificity 75% revealed that the ROMA obtained a sensitivity of 82% in premenopausal and 99% in postmenopausal women (Table 5). The specificity and the sensitivity figures for HE4 (pM) at different cut-off values and the normal CA125 (U/mL) cut-off level are shown in Table 5.

**Table 5:** Comparison of ROC–AUCs, sensitivity and specificity for ROMA, CA125 (U/ml) and HE4 (pM), discriminating malignant including borderline tumors from benign tumors

	**Premenopausal^**	**n= 123**		**Premenopausal^**		
	**AUC**	**95% CI**	**n= 123**	**cutoff**	**sens (%)**	**spec (%)**
HE4	0,73	0,58-0,87	**ROMA**	**4,6%**	82	**75**
CA125	0,82	0,73-0,92	**CA125**	**35**	**59**	68
ROMA	0,73	0,58-0,88	**HE4**	39	82	**75**
			**HE4**	**60**	59	77
			**HE4**	**70**	**41**	89
	**Postmenopausal^^**	n= 154		**Postmenopausal^^**		
	**AUC**	**95% CI**	n= 154	**cutoff**	**sens (%)**	**spec (%)**
HE4	0,91	0,87-0,96	**ROMA**	**10,4%**	99	**75**
CA125	0,91	0,87-0,96	**CA125**	**35**	**82**	84
ROMA	0,94	0,90-0,98	**HE4**	**70**	**90**	**75**
			**HE4**	100	**80**	86
			**HE4**	120	**72**	91

^Premenopausal = women < 50 year of age.

^^Postmenopausal = women > 54 year of age.

ROMA cut-off set at fixed specificity 75% as recommended by Moore et al. 2009 among patients with all types and stages of ovarian tumours.

The patient characteristics and associated preoperative HE4 levels are shown in Table 6. Patients with invasive ovarian cancer stage III-IV had significantly higher preoperative HE4 levels (median 569 pM) than those with stage 1 or 2 disease (median 101 pM, p < 0,001).

**Table 6:** Preoperative HE4 levels and characteristics of patients with epithelial ovarian cancer

	**N**	**Median**	**Range**	**p-value**
**Age**				
≤52 years	13	176.7	39.1–1032	
>52 years	72	436.9	42.8–32250	<0.05
**Histology**				
Serous	51	522.2	65.9–6248	
Non-serous	34	152.5	39.1–32250	<0.05
**Stage**				
I	28	100.7	39.1–32250	
II-IV	57	568.9	47.4–6248	<0.001
**Grade**				
3	51	587.5	42.8–32250	
1 and2	34	152.5	39.1–4682	<0.001
**Cytoreductive Surgery**				
Optimal	57	278.2	39.1–32250	
Suboptimal	28	578.2	80–6348	<0.05

Preoperative HE4 levels were significantly higher for patients with histologic grade 3 tumors (median 588 pM) than for those with grade 1 or 2 tumors (median 153 pM, p < 0,001). Patients with serous histology were more likely to have elevated preoperative HE4 levels than those with other histological types (median HE4 of 522 pM versus 153 pM, p= 0,004). The lowest median preoperative HE4 level was observed in the group of patients with mucinous ovarian carcinomas (n = 16, median 83 pM, range 42–1647, p = 0.007 ; Figure 1b).

Optimal tumor debulking (residual tumor mass ≤1 cm) after primary surgery was achieved in fifty-seven (67%) invasive ovarian cancer patients. The median preoperative HE4 level for these patients was 278 pM. The remaining twenty-eight patients (33%) had suboptimal cytoreduction and these patients had a significantly higher preoperative HE4 level (median 578 pM, p = 0.008).

The 5 years overall survival in patients with invasive ovarian cancer was 42%.

HE4 and CA125 were dichotomized at the median to discriminate between high and low risk for overall survival using univariate Cox regression analysis and Kaplan-Meier curves. In univariate Cox regression analyses high levels of HE4, CA125, increased age (continous varable), advanced-stage of disease 2–4, histological grade 3 and non-optimal tumor debulking at primary surgery were all significantly associated with shorter overall survival (Table 7). Histological types (serous histology versus other histological types) were not associated with overall survival.

**Table 7:** Cox regression analyses of overall survival

			**Univariate**	
		**HR**	**95% CI**	***p-value***
HE4	≥405 vs. <405 pM	1.9	1.1 - 3.5	0.028
CA125	≥398 vs. <398 U/ml	2.2	1.2 - 3.8	0.006
Age	continous variable	1.02	1.003 - 1.045	0.025
Stage	2-4 vs. 1	3.3	1.6 - 6.9	0.002
Grade	3 vs. 1-2	2.5	1.4 - 4.4	0.002
Residual tumor	≥1 vs. <0-1 cm	2.9	1.8 - 4.8	<0.001
**Pre-operative known parameters**		Multivariate Stepvise Backward*		
		**HR**	**95% CI**	***p-value***
HE4	≥405 vs. <405 pM	2.02	1.1 - 3.8	0.02
CA125	≥398 vs. <398 U/ml			ns
Age	continous variable			ns

*Multivariate Stepvise Backward conditional analyses, removal at 0.1 including HE4 and CA125 (dichotomized at median) and age (continous variable).

**Figure 2:**
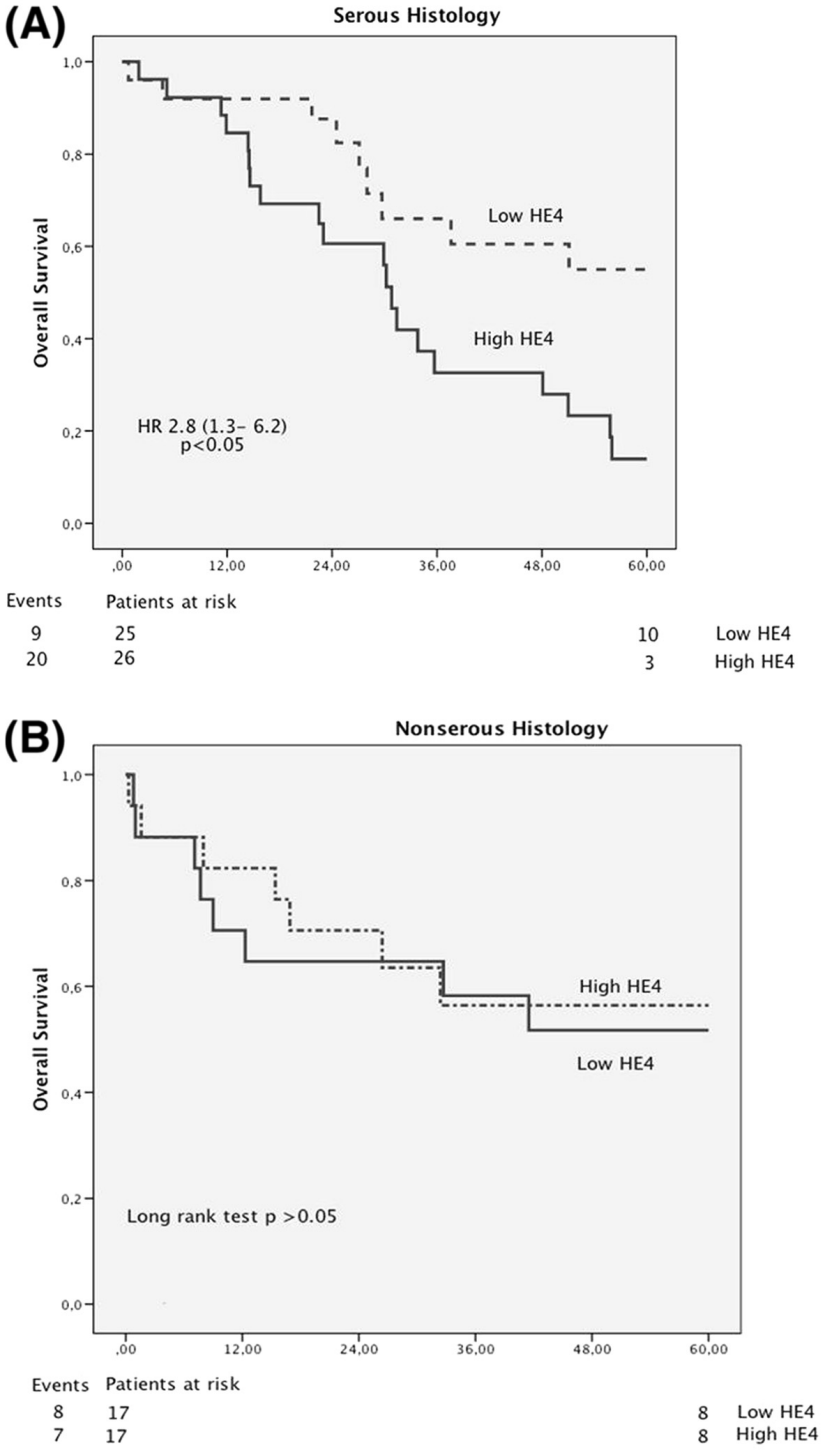
**Kaplan-Meier estimates of survival probabilities using peripheral blood concentrations dichotomized by the median of HE 4 for serous malignant tumors (A) and non-serous malignant tumors (B). **The p-value shown is the log rank statistic with the HR with 95% CI calculated using the Cox proportional hazards model. The number of patients at risk in each stratum at time 0 and 60 months after surgery are shown below the axis with the number of deaths (events) to the left.

In a separate univariate analysis with only serous cancer, higher preoperative HE4 levels were significantly associated with shortened overall survival (HR= 3.7, 95% CI 1.6-8.9, p= 0.003 ; Figure 2a). In non-serous tumors higher levels of HE4 did not predict overall survival (HR= 0.8, 95% CI 0.3-2.4, p= 0.7 ; Figure 2b).

A multivariate Cox regression model including pre-operative available covariates HE4 and CA125 both dichotomized at median in addition to age as continuous variable showed in stepwise backward analysis that high levels of HE4 was an independent prognostic marker for worse prognosis (HR 2.02, 95% CI 1.1-3.8).

## Discussion

We found the ROMA algorithm in postmenopausal women increase the accuracy to discriminate benign form malignant ovarian tumors. We also identified high plasma levels of HE4 as an independent preoperative prognostic marker for poor overall survival in multivariate analyses.

When the ROC–AUCs of the HE4 and CA125 were analyzed, these markers performed similarly in postmenopausal women but the combination of HE4 and CA125 in ROMA algorithm performed better which is in agreement with several other studies [8-14]. In premenopausal women we found that CA125 had higher AUC value than both the HE4 and ROMA. This is in contrast to earlier results by Moore et al [4], where the combination of CA125 and HE4 performed better than CA125 alone. The disparity in results is probable due to the classification of borderline tumors and to different patient populations in the published papers.

We found a gradual increase of the HE4 levels according to the loss of cell-differentiation in all histological types of ovarian epithelial carcinomas. The median plasma levels of HE4 were slightly elevated in patients with borderline tumor, surprisingly with the highest values in patients having mucinous borderline subtype. In this paper the borderline tumors were included in the malignant group. The ROMA algorithm showed a ROC–AUC of 0.94 which is a little higher AUC value than the other recent studies [14,15]. Borderline tumors are in general referred to the malignant group and some of these tumors behave in a malignant way and should be surgically removed. However, there are studies indicated that the over all survival is the same as the normal population without any further treatment in patients with borderline tumors [16,17].

HE4 was not elevated in patients with endometriosis irrespective of the extent of endometriosis or the presence of endometrioma, in contrast to CA 125. This is in agreement with studies by Huhtinen et al. [18] who revealed that HE4 serum concentrations were within normal range in patients with different stages of endometriosis. As HE4 plasma levels are not elevated in patients with endometriosis HE4 can be very useful as an additional marker to CA 125 in premenopausal women when there is suspicion of endometriosis. In premenopausal women we found HE4 increased mainly the specificity at the same sensitivity as CA125 in patients with malignancy. The high specificity of HE4 reduces the number of patients diagnosed with false positive malignancy avoiding unnecessary surgery [19]. Especially young women in reproductive age may benefit from using the ROMA score with a high predictive value for the identification of endometriosis in this patient group.

In postmenopausal women the levels of HE4 and CA125 showed similar sensitivity and specificity but the ROMA algorithm increased both sensitivity and specificity which is in agreement with several other studies [15]. In addition, in ovarian cancer patients with normal CA125 the levels of HE4 have shown to be increased in up to 50% of the women [4]. As our results indicate the ROMA algorithm performs better in patients with early-stage malignancy than CA125 alone.

We found that high levels of HE4 in the plasma samples correlated with poor survival of the patients. In fact, HE4 was an independent marker of poor prognosis in multivariate analyses of preoperative prognostic factors. A retrospective study have suggested that high preoperative HE4 levels could be associated with shorter progression-free survival in multivariate analysis [20]. In this study overall survival was chosen as the only end point, since progression-free survival is dependent on variables such as follow-up intervals and other parameters chosen to indicate progression, i.e. increased CA125, CT-scan findings, positive cytology or histopathology, or use of follow-up symptom questionnaires. We also found that preoperative HE4 levels correlated with high tumor grade and serous histology. The separate analysis with only serous or non-serous tumors indicates that the worse prognostic impact of high HE4 values is associated to the women with serous ovarian cancer. In the Kaplan-Maier curves this is also visualized as the majority of deaths during the first 16 months are in the non-serous group of women. Patients with high preoperative HE4 levels were more likely to have high-grade serous carcinomas and worse prognosis.

It is difficult to predict how advanced stage ovarian cancer will respond to neo-adjuvant chemotherapy, as the different histological subtypes of epithelial ovarian cancer show different degrees of sensitivity to platinum based chemotherapy. High-grade serous ovarian carcinomas have a higher response rate to conventional chemotherapy in comparison to low-grade serous tumors and other histological subtypes [21-23]. Patients with stage II-IV ovarian cancer mostly undergo primary surgery, and post-operative morbidity depends on how aggressive the surgery has been performed. Several studies have indicated that the residual tumor burden is the most critical factor for postoperative prognosis [23-26]. This calls for maximal effort during the primary operation, and consequently surgery has become more radical during recent years with more extensive upper abdominal surgery and bowel resections. The surgical performance is even more important in non-serous ovarian cancer since chemotherapy does not have that good response on the tumor. Since, high pre-operative levels of HE4 indicate poor prognosis mainly related to serous ovarian cancer it may be used to guide the effort of primary surgery or the possibility of neo-adjuvant surgery in combination with other pre-operative parameters. The non-serous ovarian cancer patients with lower HE4 than serous ovarian cancer probably benefit more from primary upfront surgery. A recent paper in patients with high pre-operatively CA125 levels suggests that neo-adjuvant chemotherapy followed by interval debulking surgery is associated with increased survival in patients with stage IIIc or IV disease [27]. Elderly patients especially those with intercurrent diseases and ovarian cancer who preoperatively have high HE4 in addition to high CA125 may benefit more from neo-adjuvant chemotherapy and delayed interval debulking surgery in order to minimize morbidity and not compromising overall survival.

The long-term follow up time and the same consistent treatment regimes in this study are advantages, which increase reliability. Furthermore, death among patients diagnosed with ovarian cancer is to a large extent related to progression of the malignant disease. The HE4 was analyzed in plasma in contrast to most other studies using serum but the difference in HE4 concentration did not significantly differ between plasma and serum. The Swedish Population Register, which includes all citizens, made a complete follow up of all the patients.

The ROMA algoritm including HE4 and CA125 seems to work as a diagnostic tool for malignant and borderline ovarian tumors with higher accuracy than each of these parameters separately. We also found that high plasma levels of HE4 may be used as an independent preoperative prognostic marker for poor overall survival in ovarian cancer.

## Competing interest

The authors declare that they have no competing interests.

## Authors’ contribution

Grigorios Kalapotharakos: study design; acquisition of data; analysis/interpretation of data; manuscript drafting. Christine Asciutto: study design; acquisition of data; analysis/interpretation of data; manuscript drafting. Emir Henic: providing the biobank; revising the manuscript critically. Bertil Casslén: providing the biobank; revising the manuscript critically. Christer Borgfeldt: study design; providing the biobank acquisition of data; interpretation of data; manuscript drafting, revising the manuscript critically. All authors have approved the final version of the manuscript.

## Financial support

The study was supported in part from funds from ALF, Lund University and Region Skane.
